# Appendicorectal fistula with a novel solution: First case report and review of the literature

**DOI:** 10.1002/ccr3.2380

**Published:** 2019-08-21

**Authors:** Shaun McCrystal, Joseph Kong, Peter Gourlas, Bradley Morris, Nicholas Lutton

**Affiliations:** ^1^ Division of Surgery, Department of Colorectal Surgery Princess Alexandra Hospital Brisbane Australia; ^2^ The University of Queensland Brisbane Australia; ^3^ Division of Cancer Research Peter MacCallum Cancer Centre Melbourne Australia; ^4^ The Sir Peter MacCallum Department of Oncology The University of Melbourne Melbourne Australia

**Keywords:** appendix, circular stapler, colorectal, cuff resection, fistula, rectum

## Abstract

Appendicorectal fistula can be a cause of chronic abdominal pain, forming years after an occult episode of appendicitis. It can be diagnosed with Colonoscopy and Magnetic Resonance Imaging, and successfully treated surgically with laparoscopic appendicectomy and stapled segmental cuff resection of the rectum.

## 1 INTRODUCTION

Appendiceal fistula is a rare but recognized phenomenon. Due to the uncommon presentation of appendiceal fistula affecting the gastrointestinal tract and the challenges it presents to a surgeon, we discuss the symptoms, preoperative investigations, and our surgical approach to the first reported case of an appendicorectal fistula.

Important pathologies of the appendix include acute appendicitis, appendicorectal diverticulitis, inflammatory bowel disease, and malignancy. Appendicitis is a common emergency presentation, and delayed diagnosis or untreated appendicitis can lead to perforation, abscess formation, peritonitis, and rarely death. A rare but recognized complication of acute appendicitis is the phenomenon of appendiceal fistula.

Appendiceal fistula was first described in 1846 when an appendicocutaneous fistula was discovered at autopsy.^1^ In 1957, appendiceal fistula was defined as “*the primary perforation of the appendix to an adjacent hollow viscus or to the skin*.”^2^ The hypermobility of the appendix makes it susceptible to adhering to any intra‐abdominal organ.^3^, ^4^ Various types of appendiceal fistulae have been reported including duodenum^5^; jejunum^6^; Meckel's diverticulum^7^; ileum^8^; cecum^9^; ascending colon^6^; sigmoid colon^6^; ureter^10^; urinary bladder^11^; tubo‐ovarian^12^; uterus^13^; vagina^14^, ^15^; aorta^16^; right iliac artery^17^; and cutaneous^3^ (umbilicus,^18^ right inguinal hernia,^19^ right buttock,^20^ right psoas,^21^ right loin,^22^ trauma^23^).

Reported appendiceal pathologies leading to appendiceal fistula include acute appendicitis, periappendiceal abscess, incomplete appendicectomy,^24^ malignancy (mucinous adenocarcinoma^25^), goblet cell carcinoid,^26^ isolated Crohn's disease,^27^ appendiceal diverticulitis,^28^ papillovillous adenoma,^29^ and neuroma.^30^ Reports of adjacent pathologies leading to appendiceal fistula include malignancy (cervical squamous cell carcinoma^31^); sigmoid diverticulitis^32^; Hirschsprung's disease^33^; cystic fibrosis^34^; abdominal aortic aneurysm (primary^16^ and secondary to repair^35^); arterial reconstruction^36^; and recent or previous surgery (trauma laparotomy,^23^ hysterectomy,^37^ transurethral resection of prostate,^38^ transurethral resection of bladder tumor with Mitomycin C,^39^ right inguinal hernia repair,^19^ and right groin hernia repair with propylene plug^40^).

Until now, there was no known report of a fistula occurring between the appendix and rectum. Due to the uncommon presentation of appendiceal fistula affecting the gastrointestinal tract, and the challenges it presents to a surgeon, we discuss the symptoms, preoperative investigations, and our surgical approach to the first reported case of an appendicorectal fistula.

## 2 CASE REPORT

**Figure 1:**
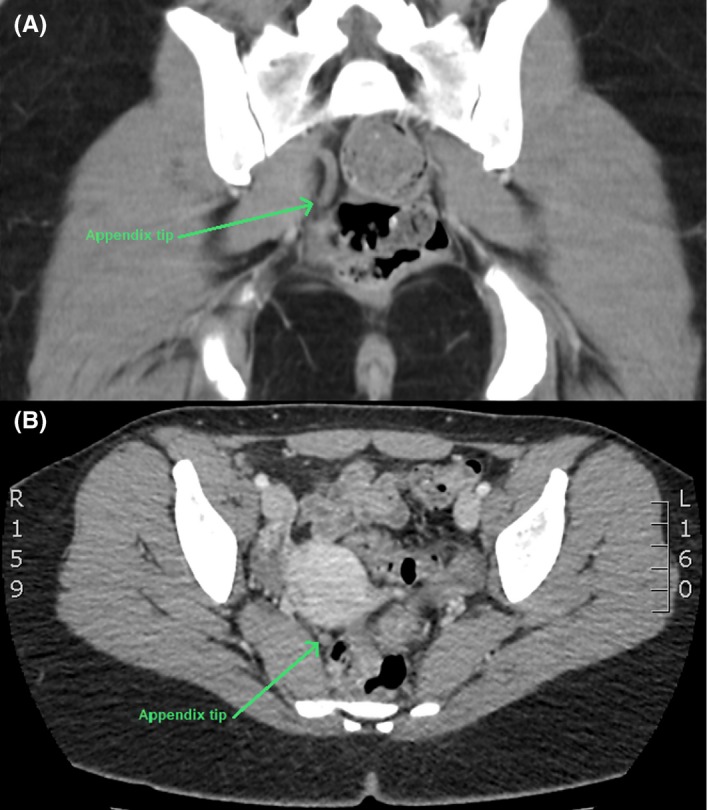
CT Abdomen: (A) Coronal View; (B) Axial View

A 23‐year‐old female experienced an episode of acute abdominal pain that led to a computed tomography (CT) scan of the abdomen. The CT revealed no intra‐abdominal pathology and her pain resolved spontaneously. However, the CT did demonstrate a long pelvic‐oriented appendix with the tip adjacent to the rectum (Figure 1). She went on to experience intermittent pelvic pain every 2‐3 months which could last up to thirty minutes at a time. Three years later, she presented to her general practitioner following an episode of severe cramping pelvic pain associated with constipation. The pain subsided after defaecation, but led to large volume diarrhea and the passage of mucous per rectum. She otherwise has no significant past history, no family history of inflammatory bowel disease and no prior colonoscopy.

**Figure 2:**
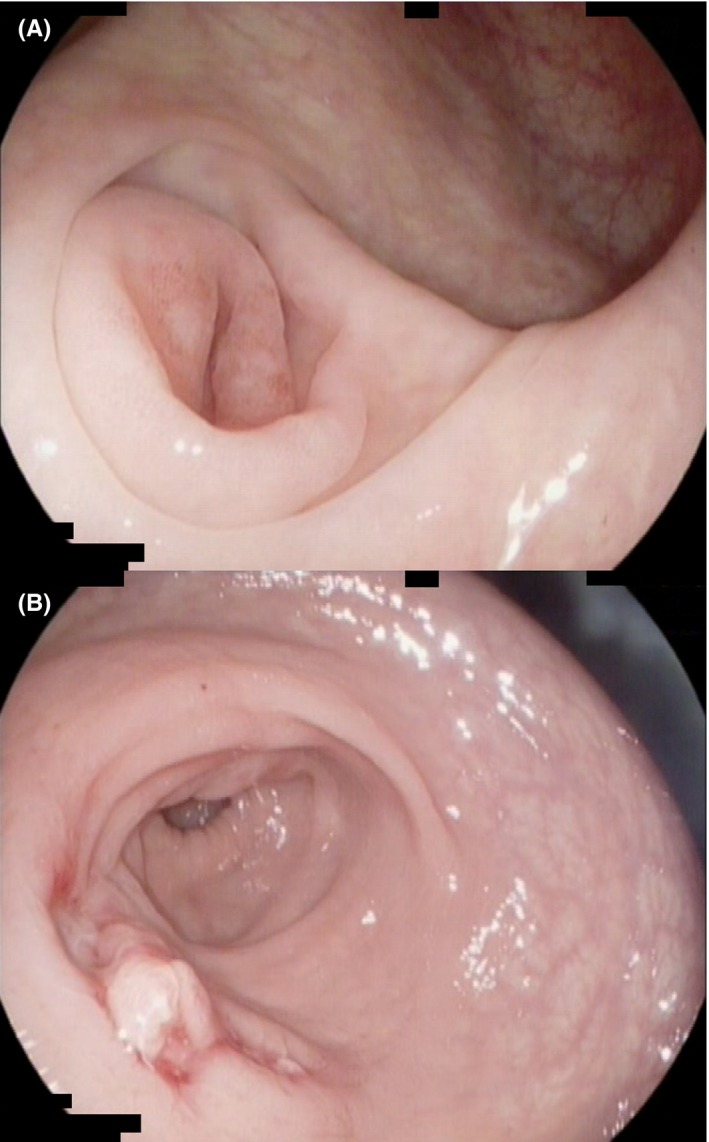
Colonoscopy: (A) Periappendicorectal Inflammation; (B) Upper Rectal Defect

**Figure 3:**
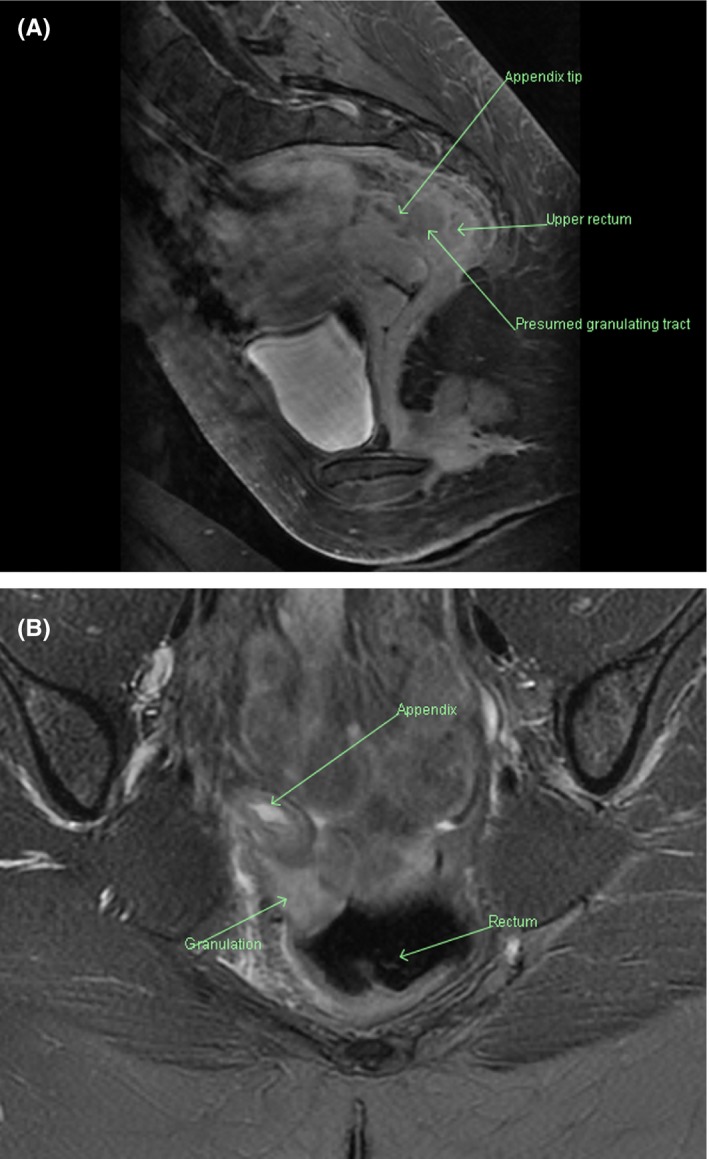
MRI Pelvis: (A) Sagittal View; (B) Axial View

Abdominal examination revealed mild lower abdominal tenderness, and rectal examination was normal. Pelvic ultrasound was negative. Proctalgia fugax was suspected. A colonoscopy was performed which demonstrated patchy inflammation around the appendiceal orifice and an upper rectal defect suspicious for a fistula. The defect appearance was similar to an open diverticulum with some mucopurulent exudate at the edges as well as a granulation polyp (Figure 2). A MRI of the pelvis confirmed a fistulous tract between the tip of a pelvic‐oriented appendix and the rectum at about the 12 cm mark (Figure 3).

**Figure 4:**
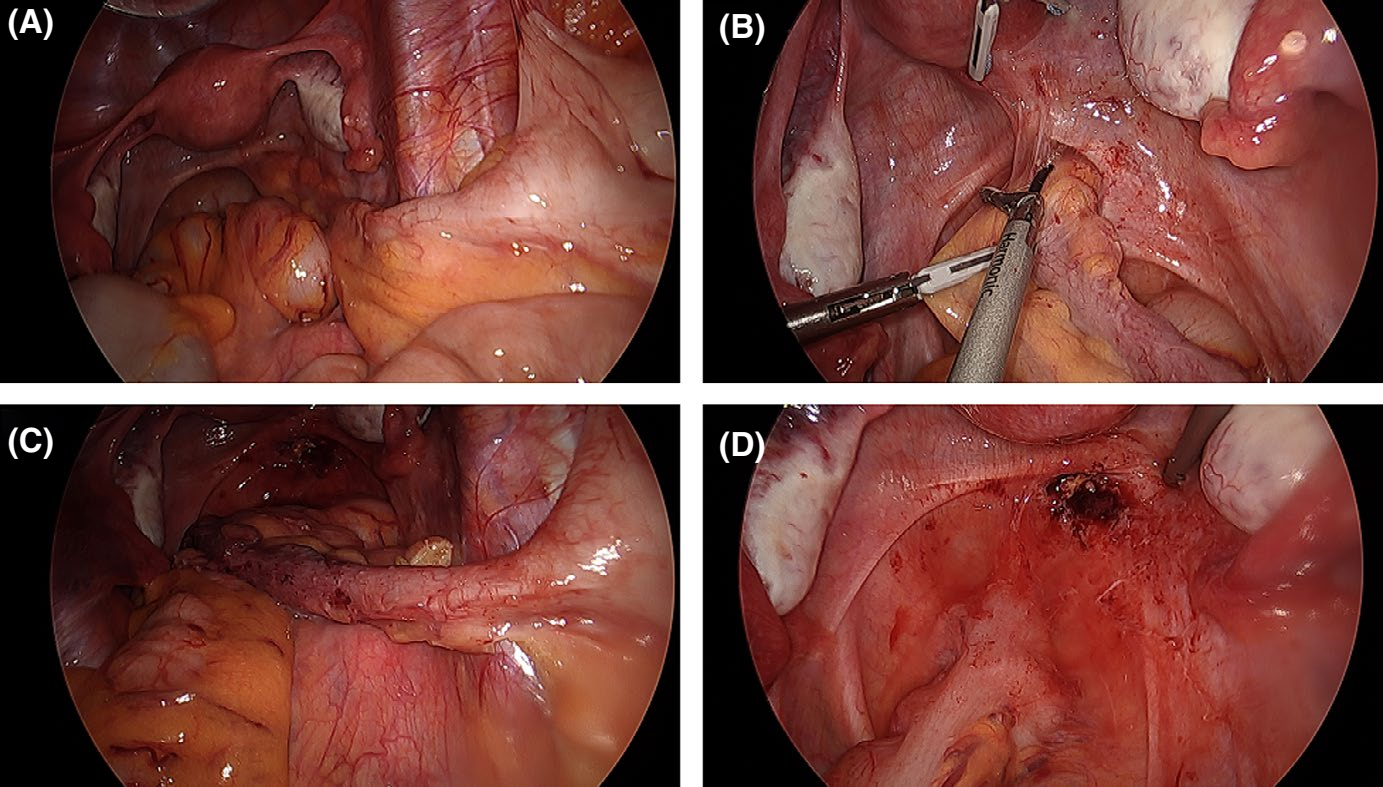
Laparoscopy: (A) Appendicorectal Fistula; (B) Dissection; (C) Separation; (D) Rectal Defect

**Figure 5:**
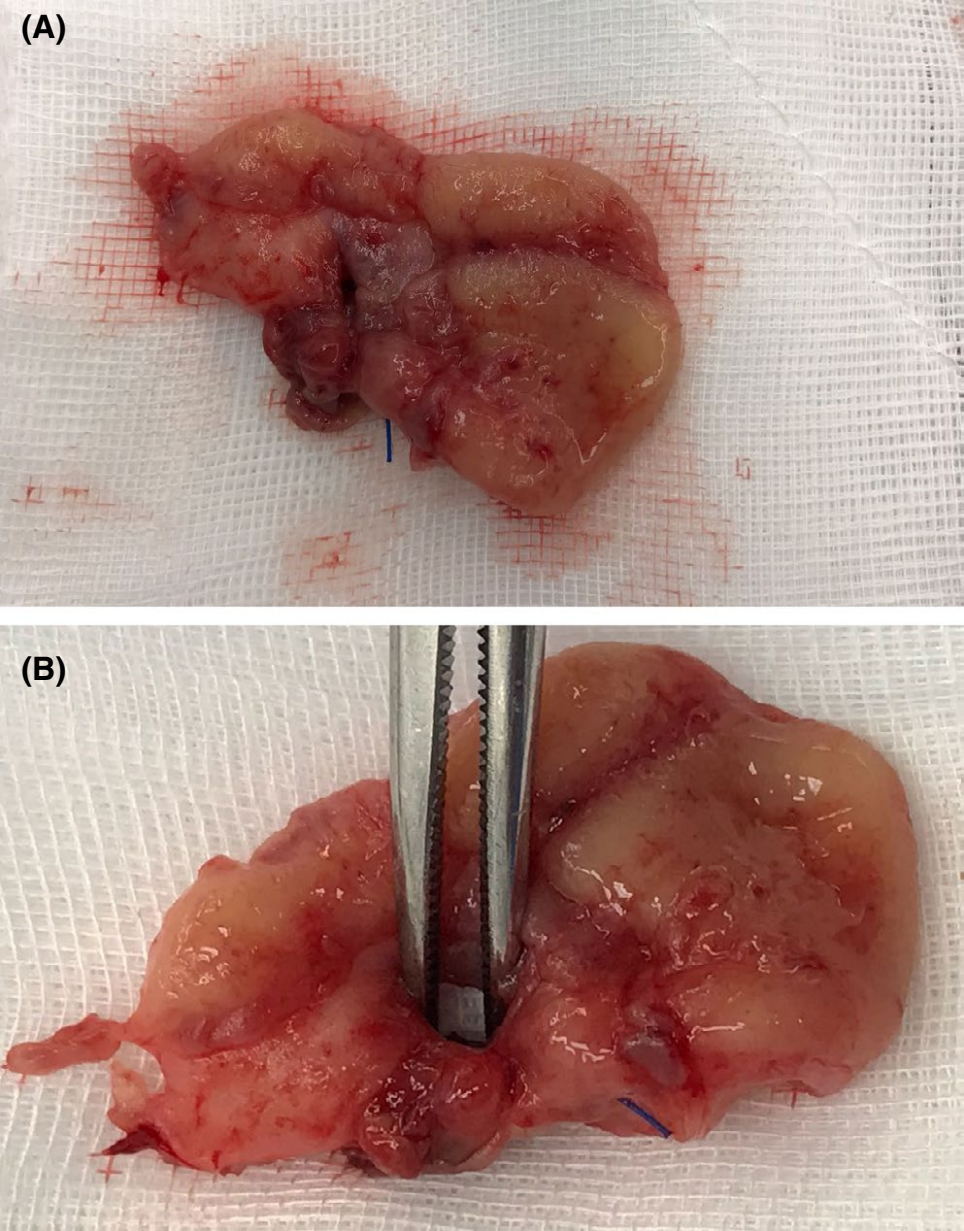
Specimen: (A) Cuff of Rectum; (B) Fistula Opening

The patient was referred for a surgical opinion. Together with her surgeon, the patient elected for surgical resection of the appendix and rectum. At the time of the operation, an 80 mm appendix was found extending from the cecum to the anterior rectum and posterior vaginal vault. The tip was dissected sharply from the rectum using an energy device, and a routine appendicectomy was performed. A defect persisted within the rectum and vaginal vault with chronic inflammatory changes around the cut edges (Figure 4). A decision was made to convert to an open Pfannenstiel incision. Small adhesions between the vaginal vault and rectum were taken down. A cuff resection of the affected anterior rectal wall was undertaken using a 29 mm intraluminal circular stapler. The cuff of rectum specimen clearly demonstrated a fistula opening (Figure 5). The staple line was reinforced with 3‐0 PDS interrupted sutures, with drain and rectal tubes placed. The rectal tube was removed on day two and the abdominal drain on day four. The patient had an uneventful postoperative course and was discharged on day five. The patient was well upon review at 4 weeks and was discharged from the outpatient clinic.

Histological examination of the appendix demonstrated features of mild acute on chronic appendicitis with crypt architectural distortion and numerous non‐necrotising granulomata and lymphoid aggregates present throughout the appendiceal wall. Granulation tissue was adherent to the serosal surface of the distal tip of the appendix and the rectal wall showed perforation with granulation tissue adherent to the deep margin extending through the wall to the mucosal surface, consistent with fistula formation.

## 3 DISCUSSION

A review of the literature demonstrates that a common presentation of “spontaneous” appendiceal fistula is chronic abdominal pain measured in years. Appendiceal fistula formation should therefore be considered in patients with chronic abdominal pain and a suspected chronic inflammatory process: a distant yet memorable episode of pain may reflect an episode of acute appendicitis. Other risk factors for appendiceal fistula include persistent or recurrent urinary tract infection; previous abdominal surgery; a chronic draining cutaneous sinus or an abscess requiring repeat incision; and drainage. There is also a notable population in patients with cystic fibrosis.^34^, ^41^, ^42^, ^43^, ^44^

Optimal surgical treatment of appendiceal fistula will of course be determined by the underlying pathology and structures involved. In our case, the investigation of a suspected case of proctalgia fugax led to the discovery of an appendicorectal fistula by colonoscopy and MRI, which may have been the sequelae of an episode of acute appendicitis on a long pelvic‐oriented appendix years earlier. As there was no evidence of malignancy, a local resection of the appendix and rectum was deemed appropriate. The closure of the inevitable defect in the rectum was achieved by novel stapled segmental cuff resection which owes its origins to and is a recognized treatment of endometriosis.^45^, ^46^

## 4 CONCLUSIONS

This is the first reported case in the published literature of an appendicorectal fistula following what appears to be an acute on chronic episode of acute appendicitis. Appendiceal fistula is a rare phenomenon that is important to diagnose and can be challenging to treat. Awareness of the types of appendiceal fistula and the various surgical options assists a general surgeon to ensure positive outcomes. Stapled segmental cuff resection of a rectal defect is a novel method of closure and treatment of appendicorectal fistula.

## CONFLICT OF INTEREST

None declared.

## AUTHOR CONTRIBUTIONS

SM: Primary author, reporting and literature searches. JK: Critical revision, final approval. PG: Critical revision, final approval. BM: Critical revision. NL: Critical revision.
